# A key role for P2RX5 in brown adipocyte differentiation and energy homeostasis

**DOI:** 10.1080/21623945.2024.2421745

**Published:** 2024-11-01

**Authors:** Maria Razzoli, Seth McGonigle, Bhavani Shankar Sahu, Pedro Rodriguez, Daniel Svedberg, Loredana Rao, Chiara Ruocco, Enzo Nisoli, Bianca Vezzani, Andrea Frontini, Alessandro Bartolomucci

**Affiliations:** a Department of Integrative Biology and Physiology, University of Minnesota, Minneapolis, MN, USA; b Cellular and Molecular Neurosciences Division, DBT- National Brain Research Center, Manesar, Gurgaon, India; c Department of Life and Environmental Sciences, Università Politecnica delle Marche, Università degli Studi di Ancona, Ancona, Italy; d Center for Study and Research on Obesity, Department of Medical Biotechnology and Translational Medicine, University of Milan, Milano, Italy; e Department of Medicine and Surgery, University of Parma, Parma, Italy

**Keywords:** Purinergic receptors, ATP, sympathetic nerves, browning, adipogenesis

## Abstract

Brown adipocytes are defined based on a distinct morphology and genetic signature that includes, amongst others, the expression of the Purinergic 2 Receptor X5 (P2RX5). However, the role of P2RX5 in brown adipocyte and brown adipose tissue function is poorly characterized. In the present study, we conducted a metabolic characterization of P2RX5 knockout male mice; next, we characterized this purinergic pathway in a cell-autonomous context in brown adipocytes. We then tested the role of the P2RX5 receptor agonism in metabolic responses in vivo in conditions of minimal adaptive thermogenesis requirements. Our data show that loss of P2RX5 causes reduced brown adipocyte differentiation in vitro, and browning in vivo. Lastly, we unravel a previously unappreciated role for P2RX5 agonism to exert an anti-obesity effect in the presence of enhanced brown adipose tissue recruitment in male mice housed at thermoneutrality. Altogether, our data support a role for P2RX5 in mediating brown adipocyte differentiation and function that could be further targeted for benefits in the context of adipose tissue pathology and metabolic diseases.

## Introduction

The brown adipose tissue (BAT) is minimally differentiated and recruited in adult humans when compared to newborns, likely due to environmental, behavioural and physiological adaptations to large body size and living at thermoneutrality in our species (i.e. preferred temperature range with minimal adaptive thermogenesis requirements) [1,2]. On the other hand, laboratory rodents are generally kept in housing conditions below their thermoneutral zone, requiring constant energy production to generate heat and to defend their body temperature [3], a fact that drives high levels of BAT differentiation and thermogenesis. Housing mice at thermoneutrality decreases the demand for adaptive thermogenesis and induces the rodent BAT to be physiologically more similar to human BAT, making mice housed at thermoneutrality a suitable model organism for understanding the role of BAT in human health and disease [4]. Accordingly, it is essential to identify BAT thermogenic programs that are inducible in conditions of minimal BAT recruitment in rodents, potentially affording to uncover mechanisms relevant to human metabolic health. This strategy has remained largely unexplored due to the lack of experimental evidence that BAT could be recruited and activated at thermoneutrality in humans and mice, aside from using β-adrenergic receptor agonists [5] or genetic manipulations impacting primary cellular functions [6].

Recent studies from several laboratories including ours demonstrate that brown adipocytes express several adenosine and purinergic receptors whose function in browning and thermogenesis is still poorly characterized [7,8]. Among the purinergic receptors, the ATP-gated ion channel P2RX5 was identified as a selective membrane marker that distinguishes brown from white adipocytes [9] with negligible expression in subcutaneous and perigonadal white adipose tissue [7]. Previous data also show that P2RX5 is the only purinoreceptor whose expression in the BAT is responsive to both environmental temperature changes and stress, either condition known to be a major stimulus of thermogenesis [7]. Furthermore, incubation of brown adipocytes with ATPγS [10,11] in the differentiation cocktail enhanced UCP1 expression in differentiated adipocytes [7], providing in vitro evidence of purinergic mediated browning in line with the known role of P2RX5 in increasing differentiation and thereby inhibiting cell proliferation [11–15]. Nevertheless, information is still lacking on how this evidence translates into functional modulation of whole-body energy metabolism.

P2RX5 deficient mice have been primarily studied for the role of this receptor in the immune system [16–18]. We sought to determine for the first time the importance of P2RX5 for metabolic and BAT thermogenic competence *in vivo*. We then characterized the role of the purinergic pathway in a cell-autonomous context for its role in browning and thermogenic function in P2RX5 receptor knockdown brown adipocytes. Finally, we demonstrated *in vivo* that purinergic signalling drives anti-obesity effects and enhances BAT UCP1 in conditions of minimally adaptive thermogenesis requirements.

## Results

### P2RX5 deficiency results in a lean and hypermetabolic phenotype in the presence of de-differentiated BAT

We first measured the expression of P2RX5 in various fat pads of male mice using qPCR and found it to be almost exclusively expressed in the interscapular BAT tissue (Supplementary Figure S1a) compared to other fat depots (F(2,10) = 15.79, *p* < 0.001). This result is consistent with results obtained by Ussar et al. [9] that demonstrated expression of this receptor at the mRNA and protein level in the BAT, but not in the perigonadal white adipose tissue (pWAT) or subcutaneous inguinal white adipose tissue (scWAT). We further confirmed the sensitivity of P2RX5 regulation to temperature changes by measuring its expression in the BAT of male mice housed either at room temperature or thermoneutrality (Supplementary Figure S1b) (*t* = 3.40, df = 16, *p* = 0.004). This result suggests a potential involvement of this receptor in BAT differentiation and thermogenesis, which is consistent with previous studies [7]. To test this hypothesis, we characterized the metabolic phenotype of P2RX5 knockout mice.

**Figure 1.**
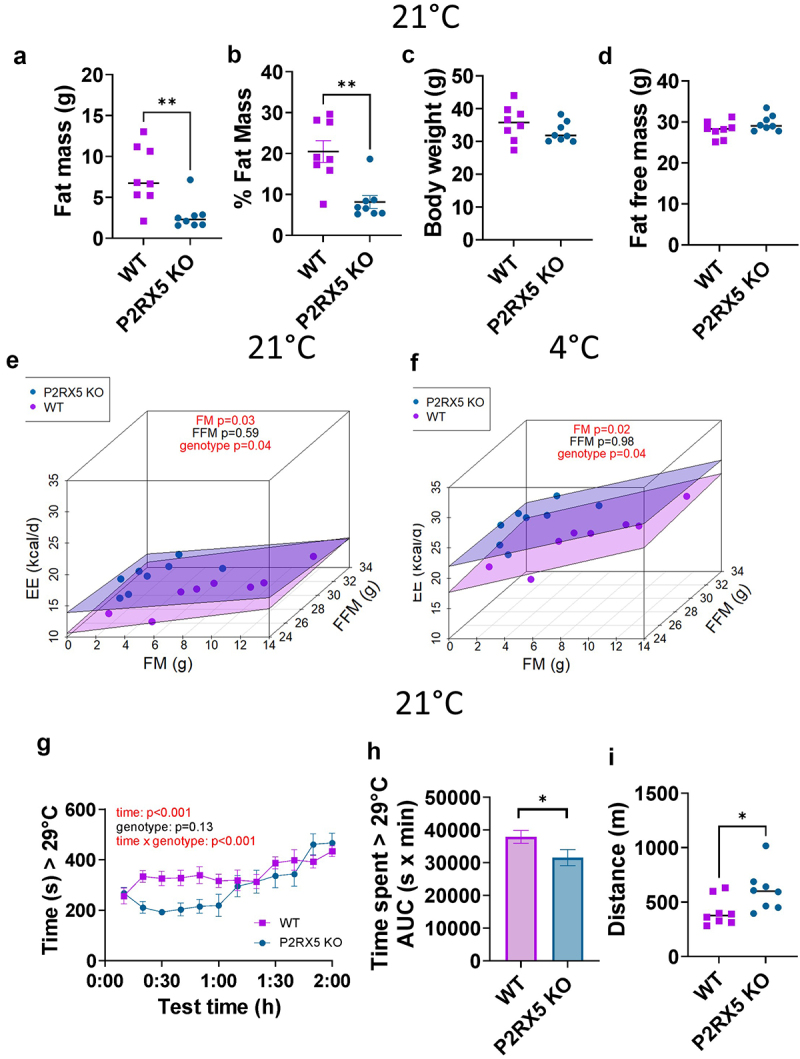
Metabolic characterization of P2RX5 knockout and wild-type mice at room temperature. (a–d) body composition: fat mass *t* = 3.34, df = 14, *p* = 0.005; % fat mass *t* = 3.99, df = 14, *p* = 0.001; body weight *t* = 1.24, df = 14, *p* = 0.23; fat free mass *t* = 1.60, df = 14, *p* = 0.13. (e–f) indirect calorimetry results illustrating energy expenditure (EE) in consideration of both fat mass (FM) and fat free mass (FFM) across 24 h at room temperature (H, fat mass F(1,12) = 5.94, *p* = 0.03; fat free mass F(1,12) = 0.30, *p* = 0.59, genotype F(1,12) = 5.10, *p* = 0.04) as well as during a 4 h cold challenge (I, fat mass F(1,12) = 7.87, *p* = 0.02; fat free mass F(1,12) = 0.001, *p* = 0.98, genotype F(1,12) = 5.09, *p* = 0.04). (g–i) results from the thermal gradient test, expressing the time spent at temperatures >29°C as intervals (G, time F(11,54 = 13.44, *p* < 0.001, genotype F(1,14) = 2.65, *p* = 0.13; time x genotype F(11,54) = 2.59, *p* < 0.001), as well as as area under the curve (AUC) (H, *t* = 1.98, df = 156,*p* = 0.049) as well as distance covered during 2 h testing (I, *t* = 2.34, df = 14, *p* = 0.03). *N* = 8/genotype. *p < 0.05, **p < 0.01.

P2RX5 knockout male mice manifest a lean metabolic phenotype at standard housing temperature (22 ± 2°C), comprising a significantly lower fat mass (*t* = 3.34, df = 14, *p* = 0.005) and proportion of fat mass (*t* = 3.99, df = 14, *p* = 0.001) than wild-type mice, while maintaining comparable body weight (*t* = 1.24, df = 14, *p* = 0.23) and fat free mass (*t* = 1.60, df = 14, *p* = 0.13) (Figure 1a–d). P2RX5 knockout mice also showed a distinct energy expenditure phenotype. After taking their body composition into account in our analysis as covariates, P2RX5 knock mice had a significantly higher energy expenditure than wild-type mice, both at room temperature (Fat mass: F(1,12) = 5.94, *p* = 0.03; Fat free mass: F(1,12) = 0.30, *p* = 0.59; Genotype: F(1,12) = 5.10, *p* = 0.04) as well as during an acute cold challenge at 4°C (Fat mass: F(1,12) = 7.87, *p* = 0.02; Fat free mass: F(1,12) = 0.001, *p* = 0.98; Genotype: F(1,12) = 5.09, *p* = 0.04) (Figure 1e,f: Supplementary Figure S3a-c). This increase in energy expenditure was not explained by locomotor activity (room temperature: F(1,13) = 0.16, *p* = 0.69; genotype: F(1,13) = 0.81, *p* = 0.38; cold challenge (activity: F(1,13) = 0.21, *p* = 0.65; genotype: F(1,13) = 0.62, *p* = 0.44) (Supplementary Figure S2a,b). Next, we used the thermal gradient test, to assess relative preference for thermoneutrality [7,19]. Interestingly, P2RX5 knockout mice showed a lower preference to occupy areas of temperature >29°C (*t* = 1.98, df = 154, *p* = 0.049) (Figure 1g-i) in the presence of significantly higher activity levels than wild-type mice (*t* = 2.35, df = 14,*p* = 0.03) (Figure 1i). This result suggests that the P2RX5 knockout mice thermoneutrality setpoint may be lower when compared to wild-type mice.

**Figure 2.**
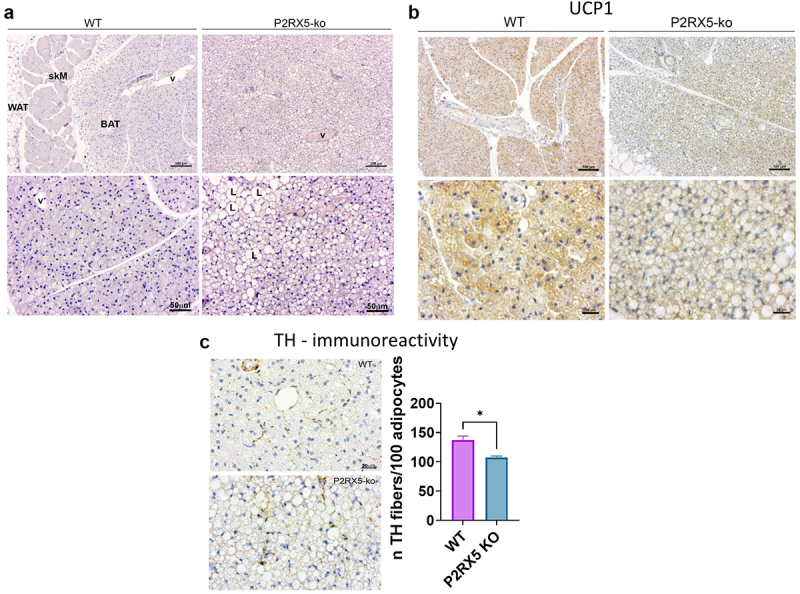
BAT histology in P2RX5 knockout and wild-type and mice. (a) Representative images of H&E staining of interscapular BAT obtained from wild-type and P2RX5 knockout mice (*n* = 3). (b) IHC performed on the same samples, disclosed a lower lever of UCP1 expression (blunted brown colour) in P2RX5 knockout mice. To note, the larger lipid droplets (L) present in brown adipocytes of knockout mice compared to wild-type (v = blood vessels; skM = skeletal muscle). See also Supplementary Figure S2c for the other tissues analysed from other WT and P2RX5 KO mice showing consistent and reproducible morphology across subjects. *N* = 3/group. (c) TH-IHC highlighting a lower density of fibres (brown spots) in the BAT of P2RX5 knockout mice. The qualitative observation was supported by morphometric evaluation confirming a significant difference in fibre density (*t* = 4.02, df = 4, *p* = 0.02). Scale bar in top rows of A and B = 100 µm. Scale bar in the bottom rows of A = 50 µM and B = 20 µm. Scale bar in C = 20 µm.

We next analysed the morphology of the adipose fat pads. Based on the lean and hypermetabolic phenotype, we hypothesized that the BAT would be more activated in P2RX5 knockout mice compared to the wild-type. Contrary to this hypothesis, visual inspection of the BAT of three mice per genotype showed the classical multilocular morphology and UCP1 level in wild-type mice (Figure 2a,b; Supplementary Figure S2c), while brown adipocytes of P2RX5 knockout mice showed a larger proportion of paucilocular or unilocular lipid droplet accumulation with reduced immunohistochemical staining of UCP1 (Figure 2a,b; Supplementary Figure S2c). Furthermore, the spot-like pattern of TH-IHC staining in P2RX5 knockout mice samples appeared to be reduced, and the quantification by morphometric evaluation revealed a significantly lower density compared to wild-type mice (Figure 2c).

In scWAT, the morphological changes were less evident with slightly smaller adipocytes found in the peri-lymph node area in P2RX5 knockout mice where multilocular adipocytes were not visible when compared to wild-type mice (Supplementary Figure S2d).

Altogether the higher energy expenditure of P2RX5 knockout mice, preference for sub thermoneutral temperatures, hyperactivity, and leaner body mass than wild-type mice – when considered together with the evidence of decreased BAT function, suggest a compensatory increase in basal metabolic rate, that is independent of BAT function.

### Cell-autonomous effect of P2RX5 knockdown in brown adipocytes

**Figure 3.**
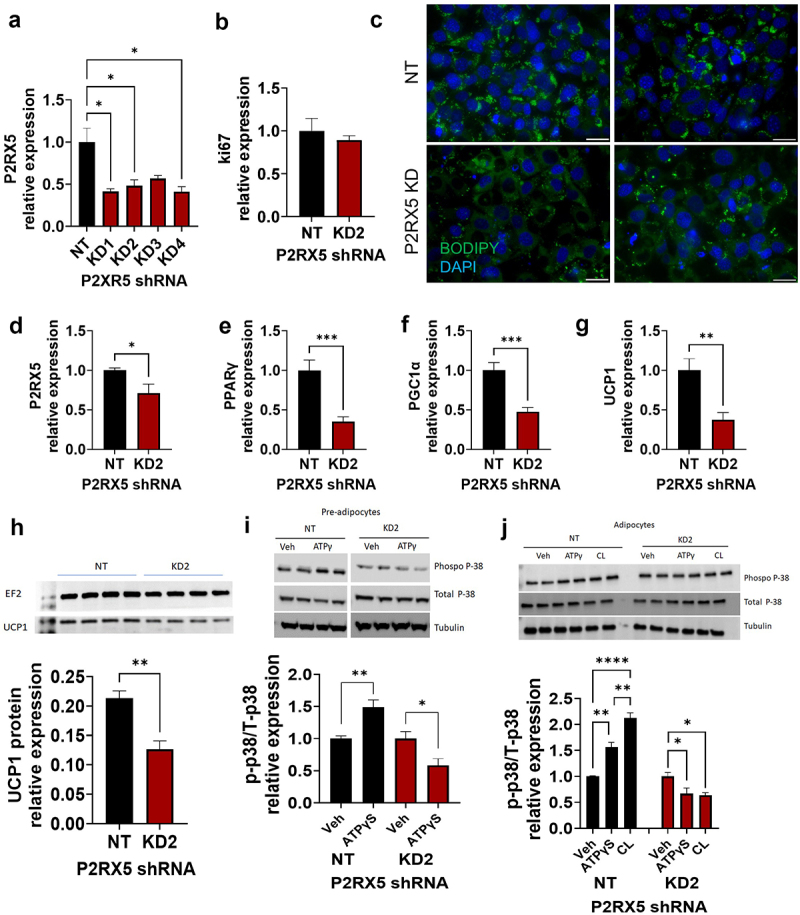
P2RX5 knockdown in brown adipocytes. (a) shRNA KD validation (Kruskal-Wallis = 9.3, *p* = 0.0192) shRNA 2 (KD 2) was selected for further analyses (b). Ki67 expression in non-targeting control (NT) and P2RX5 KD2 shRNA (t = 0.56, df = 3, *p* = 0.31). (c) BODIPY staining in NT and P2RX5 KD2 shRNA (representative of N = 4; scale bar = 30 µm). d−g) gene expression in differentiated adipocytes. (d) P2RX5 expression in NT and P2RX5 KD2 shRNA (t = 2.423, df = 8, *p* = 0.042) (e) PPARγ expression in NT and P2RX5 KD2 shRNA (t = 4.570, df = 8, *p* = 0.0009). (f) PGC1α expression in NT and P2RX5 KD2 shRNA (t = 4.673, df = 8, *p* = 0.0008). (g) UCP1 expression in NT and P2RX5 KD2 shRNA mRNA (t = 3.649, df = 8, *p* = 0.0033). (h) UCP1 protein (t = 4.61, df = 6, *p* = 0.002) with representative immunoblots in NT and P2RX5 KD2 conditions (the unedited blots are in supplementary figure S4). (i) Representative immunoblots and relative expression of phospho-*p*-38 and total p38 in the vehicle and ATPγS in NT (t = 4.180, df = 6, *p* = 0.0029) and P2×R5KD shRNA (t = 2.787, df = 6, *p* = 0.0317) in pre-adipocytes (the unedited blots are in Supplementary figure S5). (j) Representative immunoblots and relative expression of phospho-*p*-38 and total p38 in the vehicle, ATPγS and CL316,243 (CL) in NT (F_2,8_ = 41.93, *p* = 0.0001) and P2×R5KD (F_2,9_ = 6.515, p = 0.0178) shRNA in adipocytes (the unedited blots are in Supplementary figure S6). N = 4-5 from 2 independent experiments. *p < 0.05, **p < 0.01, ****p < 0.0001.

To further elucidate the role of P2RX5 in brown adipocytes in a cell-autonomous manner, we silenced its expression in immortalized brown pre-adipocytes previously generated by our laboratory [7]. P2RX5 knockdown (KD) was generated in four independent cell lines, all showing various degrees of gene downregulation (Figure 3a). KD2 was selected for further analyses. P2RX5 KD did not affect proliferation (assessed via Ki67, Figure 3b). Differentiation was slightly blunted (Figure 3c), P2RX5 expression decreased (Figure 3d) and associated with a reduction in PPARγ and PGC1α mRNA expression levels in the P2RX5 KD adipocytes compared to NT controls (Figure 3d,e). UCP1 mRNA (Figure 3f) and protein (Figure 3g) were also downregulated).

To investigate the mechanism of P2RX5-mediated differentiation, control and P2RX5 KD cells were incubated with the beta 3 adrenergic receptors (β3AR) agonist CL316,243 or ATPγS, a hydrolysis-resistant P2RX agonist that has been extensively used to test the function of P2RX5 [10,11,20,21]. ATPγS was used because of the lack of selective P2RX5 agonists. Phosphorylation of p38, the critical node for UCP1 expression and browning [22,23], was significantly increased by the γ3AR agonist CL316,243 in NT control cells (Figure 3h,i), with ATPγS inducing a similar – albeit lower in magnitude when compared to CL316,243 – increase in phosphorylation of p38. Conversely, P2RX5 KD cells manifested defective CL316,243-induced and ATPγS-induced p38 phosphorylation which is in line with blunted differentiation. These data support a key role for P2RX5 in ATPγS-induced brown adipocyte differentiation which is consistent with other cell types [14,16]. Our data also support an ATP/P2RX5 mechanism of p38 activation mediating brown adipocyte differentiation.

### P2RX5 agonism induces BAT browning and anti-obesity effect under minimal adaptive thermogenesis requirements

**Figure 4.**
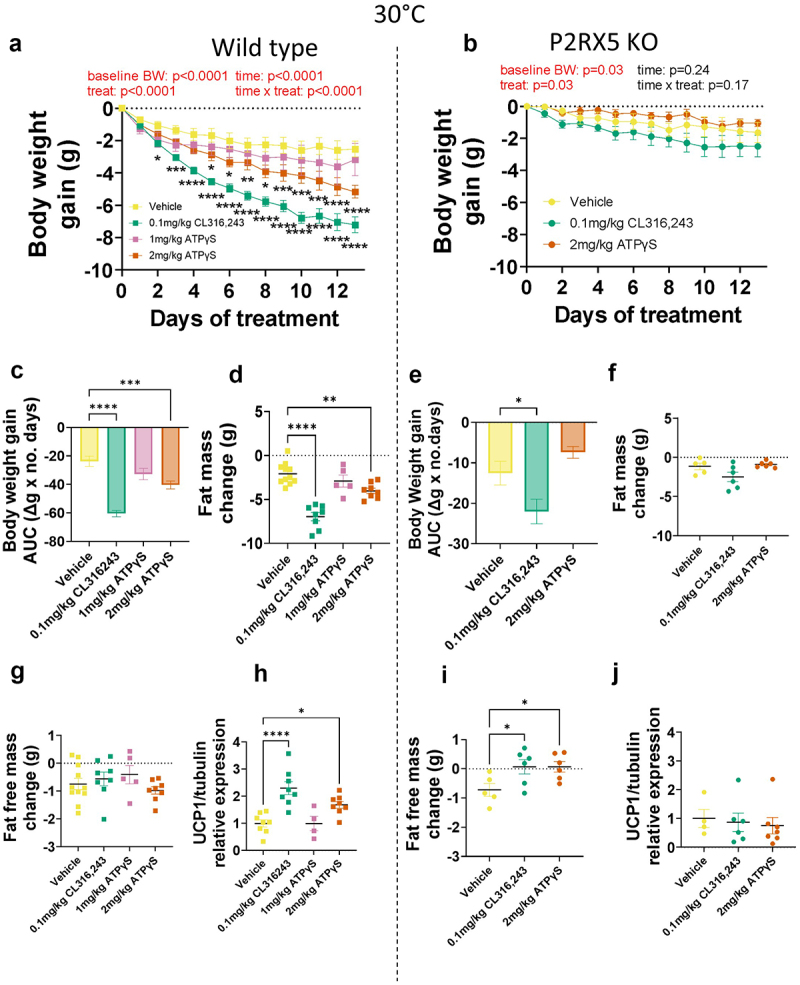
Pharmacological characterization of P2RX5 knockout (KO) (*N* = 5-6/treatment) and wild-type mice (*N* = 5-11/treatment) to adrenergic and purinergic agonism at thermoneutrality (30°C). (a–b) body weight change after a month of acclimation to 30°C during 13 daily treatment injections. (c–e) body weight area under the curve during the pharmacological treatment. (d–f) fat mass change and (g–i) fat free mass change during the pharmacological treatment. h-j) protein expression of UCP1 over beta-tubulin loading control relative to the vehicle group. Western Blot images can be found in Supplementary Figure S7. *p < 0.05, **p < 0.01, ****p < 0.0001.

Our *in vivo* and *in vitro* loss of function studies suggest that P2RX5 is implicated in brown adipocyte differentiation. Previous work indicates that P2RX5 pharmacological activation can increase UCP1 expression in vitro [7]. Thus, to test if engagement of P2RX5 *in vivo* could exert beneficial metabolic effects, we treated P2RX5 knock out and wild-type mice with a β3AR agonist or purinergic agonist ATPγS. Mice were housed at 30°C to minimize adaptive thermogenic requirements and BAT differentiation and subsequently received 13 daily injections of the β3AR agonist CL316,243 (0.1 mg/kg) or ATPγS (1 or 2 mg/kg). As expected [5,24,25], CL316,243 treatment significantly reduced body weight (main effect treatment: F(3,412) = 24.72, *p* < 0.0001; Figure 4a–c), and fat mass (main effect treatment: F(3,28) = 25.58, *p* < 0.0001; Figure 4d), and increased BAT UCP1 (main effect treatment: F(3,24) = 113.29, *p* < 0.0001; Figure 4h; Supplementary Figure S7a), without affecting fat free mass (main effect treatment: F(3,28) = 1.00, *p* = 0.41; Figure 4g) and food intake in wild-type mice (Supplementary Figure S3d). Surprisingly, 2 mg/kg ATPγS exerted a similar effect as CL316,243 with reduced body weight and fat mass and increased BAT UCP1 (Figure 4a–c,d-h). The 2 mg/kg ATPγS dose also reduced food intake in WT mice (main effect treatment: F(3,24) = 113.29, *p* < 0.0001; Supplementary Figure S3d).

In P2RX5 knockout mice the anti-obesity effect of CL316,243 was vastly reduced, and limited to a decrease in body weight gain (F(2,218) = 8.90, *p* = 0.0002) and increased fat free mass gain (F(2,14) = 4.09, *p* = 0.04, while ATPγS (2 mg/Kg) was completely ineffective (Figure 4b–e,f–i; Supplementary Figure S3e). Finally, neither CL316,243 or ATPγS increased UCP1 in the BAT of P2RX5 KO mice (F(2,14) = 0.15, *p* = 0.86) (Figure 4j; Supplementary Figure 7d).

These data support a mechanism whereby ATPγS can increase BAT browning and exert a beneficial metabolic effect in mice housed at thermoneutrality, with a mechanism requiring P2RX5 expression, that is potentially more substantial than previously thought given the blunted thermogenic and metabolic responses seen in P2RX5 knockout mice.

## Discussion

The present results show through loss and gain of function approaches that P2RX5 are crucial regulators of brown adipocyte differentiation and function. P2RX5 deficiency caused a whitening of the BAT in vivo in male mice and a blunted differentiation of brown adipocytes in vitro. Furthermore, pharmacological treatment with a P2RX5 agonist caused browning and a beneficial metabolic effect in wild-type male mice housed at thermoneutrality, which was prevented by P2RX5 deletion.

Thus, P2RX5 receptors fulfill all requirements to be used as potential drug targets for brown adipocytes, based on their localization on the cell membrane and specificity compared to more than 40 other tissues in both mice and humans [9]. Furthermore, our previous work identified a functional purinergic system in the human BAT [7]. Nevertheless, historically there has been limited interest in studying this P2X subtype due to the canonical human P2RX5 being non-functional due to truncation (hP2X5A, 422 aa), missing the critical peptide binding sequence (22 aa) encoded by exon 10 [20]. More recently a low proportion (around 10%) of humans were found to possess full-length P2RX5 subunits (444 aa) and can form competent P2RX5 [21]. So far, the full-length P2RX5 has been identified in a small ethnically defined group [26] but may occur in a wider population as more ethnicities are screened, a discovery that should prompt a re-evaluation of the druggability of this receptor. Therefore, it is even more critical to expand the knowledge of the physiological function of the P2RX5 receptor, especially considering the prominence of obesity [27,28].

P2RX5 receptors are widely distributed in murine tissues, where they play a supporting role in the inflammatory response [21]. However, previous data [9], and present results demonstrate that in mouse adipose fat pads gene expression is essentially restricted to the BAT, with expression in other fat pads being negligible. Genetic deletion of P2RX5 has been shown to decrease inflammatory bone loss, and expression levels of pro-inflammatory cytokines compared with wild-type mice [16,29]. P2RX5 knockout has also been associated with mild behavioural and neurological phenotypes [30] and jax.org, MGI:6263086), but no previous metabolic characterization was available. Here we showed that P2RX5 knockout mice possess a paradoxical metabolic phenotype. Germline P2RX5 deletion corresponded to an enhanced thermogenic phenotype in mice housed at standard temperatures which became more apparent after mice were cold challenged. This phenotype was not associated with hyperlocomotion or increased BAT browning, suggesting enhanced basal thermogenesis. P2RX5 knockout mice were previously reported to possess an increased level of exploratory behaviour in a novel environment [30] and jax.org, MGI:6263086). This observation was confirmed in the present study; however, their hyperactivity did not explain the enhanced energy production, possibly resulting instead from a higher basal metabolic rate as suggested by their preference for lower ambient temperatures. This conclusion was further supported by the morphological inspection of the BAT showing a higher lipid accumulation, a lesser sympathetic fibre innervation, and UCP1 expression, altogether supporting the notion of an impaired thermogenic capacity of the BAT in P2RX5 knockout mice.

To increase the representative value of mouse models to human thermogenic function and to study the thermogenic contribution of pathways other than the canonical adrenergic pathway, we minimized thermogenic requirements – and BAT differentiation – by housing mice at thermoneutrality [7,31]. Mice were subsequently chronically administered either with the classic β_3_-AR agonist CL316,243, or the purinergic receptor agonist ATPγS. In this condition, an anti-obesity effect became evident in wild-type mice following either drug regimen that resulted in comparable levels of weight loss and BAT browning as suggested by increased UCP1. On the other hand, in P2RX5 knockout mice, the purinergic stimulation was ineffective, and even the anti-obesity effect of the β_3_-AR agonist was blunted. The lack of efficacy of ATPγS in P2RX5 knockout suggests that its browning effect is primarily mediated by P2RX5. However, the only marginal efficacy of CL316,243 in P2RX5 KO male mice points again to a diminished differentiation and thermogenic competence of the brown adipocytes caused by P2RX5 deficiency; a result consistent with our *in vitro* characterization of P2RX5 knockdown in brown adipocytes. Indeed, although the P2RX5 knockdown cells were able to proliferate similarly to wild-type cells, their ability to differentiate was blunted, as demonstrated by lower expression levels of markers such as PPARγ, PGC1α, and UCP1 [14,16]. Furthermore, P2RX5 knockdown cells also showed decreased levels of β3AR-mediated p38 MAPK phosphorylation, suggestive of impaired thermogenic functions [23]. Control and P2RX5 knockdown cells were also acutely incubated with ATPγS to investigate the mechanisms of ATP-induced differentiation. ATPγS induced a functional response in both pre-adipocytes and adipocytes of the NT cells, albeit of a greater magnitude in the former, since pre-adipocytes express lower levels of P2RX5 than mature adipocytes [7,9]. This effect was entirely prevented and significantly reduced in P2RX5 knockdown cells.

Our in vitro and in vivo experiments suggest that the effect of P2RX5 deletion causes its effects in a cell-autonomous manner in brown adipocytes. However, since we used germline knockout mice we cannot entirely rule out the possibility that the BAT tissue dysfunction could be in part caused by physiological changes in other cell types involved in thermogenesis such as the preoptic area and hypothalamus [32], and immune cells [21,33] including macrophages [33,34]. Similarly, although P2RX5 KO abolished the effects of ATPγS on body weight and browning, we cannot rule out that ATPγS could modulate purinergic receptors in sympathetic neurons which express P2RX1–4 receptors [35] or other cell types. Brow adipocytes respond to adrenergic stimulation by increasing membrane conductance [36], elevating intracellular calcium concentration [37–39], and activating thermogenesis [40]. Purinergic stimulation by ATP also increases intracellular calcium concentration and initiates heat production [41]. In addition, ATP but not noradrenaline increases membrane trafficking [42]. Previous data suggest that adrenergic and purinergic receptor stimulation may converge in altering the function of voltage-gated K^+^ currents affecting brown adipocyte proliferation [43,44]. The properties of the K^+^ channels indicate that they could have a role in producing an increase in K^+^ permeability of the brown fat cell membrane during the depolarization that accompanies NE-stimulated thermogenesis [45], but no direct evidence that this might be the case exists yet. Following this logic and in considering our results, future studies should contemplate co-administration strategies of the two receptor agonists, for efficacy in treating obesity in mouse models.

One limitation of our in vivo study is that mice were bred in homozygosity which may lead to cage effects on development, behaviour and physiology [46]. Thus, future studies are required to confirm the present results in mice derived from heterozygous parents, and raised as mixed genotypes littermates, and extend the investigation to female mice that were not tested here. Nevertheless, this limitation is mitigated by the substantial consistency between the BAT and metabolic phenotype of the P2R5 KO mice, and the cell-autonomous effect conferred by P2RX5 KD in brown adipocytes in vitro.

Altogether our data show for the first time a metabolic role for P2RX5 in modulating brown adipocyte differentiation and energy balance. Furthermore, we showed that modelling human energy metabolism via thermoneutrality housing in mice can be a promising strategy for identifying therapeutic approaches to increase BAT differentiation. Using this strategy, a pro-browning and anti-obesity role for purinergic agonism became evident in mice. These data should promote future studies to therapeutically explore the adjunct benefit of a pro-differentiation pathway to BAT functions and its anti-obesity potential.

## Materials and methods

### Mice

P2RX5 KO mice were maintained in our colony derived from breeders generated by Y. Choi (University of Pennsylvania) on a C57BL/6J background using P2RX5 knockout sperm obtained from the International Mouse Strain Resources (IMSR) [16]. Mice were maintained in a fully controlled animal facility (12:12 h light:dark cycle at 22 ± 2°C). Homozygous breeding pairs were established, and pups were weaned in groups of same-sex and same-genotype siblings. Adult C57BL/6J (JAX#000664) male mice were purchased from Jackson Labs to serve as wild-type controls. All mice were genotyped according to a previously established protocol [16]. All mice were maintained and used in accordance with guidelines approved by the Institutional Animal Care and Use Committee (IACUC) at the University of Minnesota. Mice were fed a standard chow diet ad libitum (Teklad Global 18% Protein Rodent Diet). We adhere to the ARRIVE guidelines.

### Experiment 1: metabolic characterization of P2RX5 knockout mice

P2RX5 knockout (*n* = 8) and wild-type (*n* = 8) male mice were tested in the thermal gradient test (TGT). TGT was conducted by placing an individual mouse in a 120 cm long polypropylene carbonate corridor with a temperature-controlled aluminium floor to form a floor temperature gradient from 21°C to 33°C. Mouse occupancy across the corridor was monitored via a video camera and acquired using the video tracking software Ethovision XT (Noldus, The Netherlands) for 2 hours. A week later they were tested for body composition with Echo MRI 3-in-1 (Echo Medical System) and energy expenditure via indirect calorimetry. Mice were maintained in the indirect calorimetry system for 3 d, consisting of a 24 h acclimation phase and a 24 h acquisition phase at 22 ± 1°C, followed by 24 h of exposure to 4 ± 1°C. Oxygen consumption (VO_2_) and carbon dioxide production (VCO_2_) were measured using the Oxymax Comprehensive Lab Animal Monitoring System (Columbus Instruments). Energy expenditure was calculated using the formula provided by the manufacturer, expressed as Kcal/h and fat mass and fat free mass were analysed as continuous predictors in an ANCOVA model [47].

A subset of P2RX5 knockout (*n* = 3) and wild-type (*n* = 3) mice housed at 22 ± 1°C were sacrificed, immediately tissue pieces representing brown adipose tissue (BAT) and subcutaneous inguinal adipose tissue (scWAT) were harvested and fixed in 4% paraformaldehyde in 0.1 M phosphate buffer (pH 7.4) by overnight immersion at 4°C. The samples were then dehydrated, cleared, and embedded in paraffin. Serial sections, from 3 different levels (100 μm apart), were respectively haematoxylin and eosin-stained to assess their morphology and immuno-stained for uncoupling protein 1 (UCP1) and tyrosine hydroxylase (TH). Immunostaining was performed as follows: 3-μm-thick sections were dewaxed and, incubated with anti-UCP1 (1:500; ab10983, Abcam, Cambridge UK) or anti-TH (1:300; AB1542 Merck KGaA, Darmstadt, Germany) according to the avidin-biotin complex (ABC). Briefly: 1) endogenous peroxidase blocking with 3% hydrogen peroxide in methanol; 2) normal serum blocking (1:75) for 20 min to reduce non-specific background; 3) incubation with primary antibodies against UCP1 or TH overnight at 4°C; 4) secondary antibodies specific for each species in which the primary antibody was raised, IgG biotin-conjugated (1:200; Vector Labs, Burlingame, CA, USA); 5) ABC kit (Vector Labs); and 6) enzymatic reaction to reveal peroxidase with Sigma Fast 3,3’-diaminobenzidine (Merck KGaA, Darmstadt, Germany) used as substrate. Finally, sections were counterstained with haematoxylin and mounted in Eukitt (Fluka, Deisenhofen, Germany). For the evaluation of TH-immunoreactive fibre density, we collected random pictures using a 100 × oil immersion objective to reach 100 adipocytes for each animal (*n* = 3) of the two genotypes. We then counted the total number of TH-IR fibres. All observations were performed with a Nikon Eclipse 80i light microscope (Nikon, Tokyo, Japan) equipped with a CCD camera. The brightness and contrast of the final images were adjusted using the Photoshop CS3 software (Adobe Systems; Mountain View, CA, USA).

### Experiment 2: generation and characterization of P2RX5 knockdown brown adipocytes

Immortalized brown pre-adipocytes were previously generated [7]. These cells differentiate well with standard differentiation media and have been substantially validated [7]. Stable P2RX5 KD was generated in these cells. Cells were cultured in DMEM + 10% FBS, plated at 30% confluency and transfected with Sure silencing shRNA plasmids (four different sequences) KM31857H (Qiagen) by using lipid-based transfection reagent (MIRUS BIO). Seventy-eight hours after transfection, cells were cultured in a medium with hygromycin (1 mg/ml) and stably selected. Cells were cultured in the selection media for at least 20 d and gene expression was quantified using qPCR. Bodipy (1:3000 dilution) and DAPI (0.5 ug/mL) were used for imaging. For the experiments described in Figure 3, undifferentiated and differentiated cells were treated with ATPγS (15 min, 10 nM), or CL316,243 (10 min, 10 nM).

### Experiment 3: metabolic characterization of purinergic activation in conditions of minimal adaptive thermogenesis in wild-type and P2RX5 knockout mice

P2RX5 knockout and wild-type male mice were acclimated to 30 ± 1°C for a month before starting the procedures. Mice (*n* = 5-11/genotype/group) received 13 daily i.p. injections of either ATPγS (1-2 mg/kg; A1388 Millipore-Sigma), CL316,243 (0.1 mg/kg; NC1408675 Fisher Scientific) or sterile saline. Mouse body composition was assessed before starting the injections and after the last injection. Body weight and food intake were measured daily.

### Western Blot

Proteins were extracted from frozen BAT in RIPA buffer (50 mm Tris – HCl, 150 mm NaCl, 1 mm EDTA, 0.5% Triton X-100, 1% sodium deoxycholate, 0.3% SDS, 0.1 mm PMSF, 0.2 mm 1, 10-phenoanthroline monohydrate, Phosphatase Inhibitor Cocktail A (Sigma – Aldrich, St. Louis, MO), Protease Inhibitor Cocktail (Sigma – Aldrich), and Phosphatase Inhibitor Cocktail 2 (Sigma – Aldrich, St. Louis, MO) by homogenization in Precellys CK28 Lysing Kit (Bertin, Catalog # 10144–494) using a BeadBlaster 24 R Homogenizer (Benchmark Scientific, D2400-R). Homogenates were centrifuged for 30 min at 21,000 g at 4°C, with the supernatant collected and transferred to a new tube. The supernatant was centrifuged again at 21,000 g at 4°C for 10 min, and the resulting supernatant was collected. Protein concentration for each sample was determined using the Pierce BCA Protein Assay Kit (Thermo Fisher Scientific, Waltham, MA). Equal amounts of protein (35ug) were loaded into 4–20% Bio-Rad Mini-Protean TGX Precast gels (Bio-Rad, Hercules, CA). Proteins were transferred to a nitrocellulose membrane (Bio-Rad, Hercules, CA) using a Bio-Rad Trans-Blot Turbo Transfer System machine. Transferred membranes were blocked with 5% Non-Fat dry milk (Cell-Signaling, Danvers, MA) in 1 × TBST buffer (10 mm Tris-Base (Sigma – Aldrich, St. Louis, MO), 0.2 m NaCl (Macron Chemicals), 0.1% Tween-20 (Sigma – Aldrich, St. Louis, MO), with a pH 7.4. Proteins were probed overnight at 4°C with primary antibodies Anti-beta Tubulin and Anti-UCP1 (Ab6046 Rabbit polyclonal to beta Tubulin, Ab10983 Rabbit polyclonal to UCP1, Abcam, 1:2000 dilution in 2.5% Non-Fat milk in 1× TBST buffer). After primary antibody incubation, the membrane was incubated with secondary antibody Goat Anti-Rabbit IgG H&L (HRP) (Ab205718, Abcam, 1:15,000 dilution in 2.5% Non-Fat milk in 1× TBST buffer). The ladder we used is from LI-COR (Selected P/N: 926–98000). Reactivity was imaged using LiCor Imaging System (LI-COR Biosciences, Lincoln, NE), and immunoreactivity was quantified using LiCor Imaging software. A similar protocol was followed for cell lysates except for loading 20 µg protein per condition. Antibodies used for the cell lysate were UCP1 (Pa1 -24,894 Rabbit polyclonal Thermofisher), Phospho-p38 MAPK (Thr180/Tyr182) Antibody #9211, and p38 MAPK Antibody #9212 (Thermofisher), EF-2 (C-9) sc -166,415 from Santa Cruz Biotechnologies. The dilution of the antibodies was: 1:2000 for beta-tubulin and 1:1000 for both total p38 and phosphorylated-p38.

### Statistical analyses

Statistica (version 14.0.1.25, TIBCO Software Inc.) and GraphPad Prism (version 10.1.2, GraphPad Software LLC) were used for all analyses. The data are expressed as means ± standard error (SD). Multiple comparisons following a one-way analysis of variance were performed using Tukey’s HSD or Dunnett’s post hoc tests. A comparison between the two groups was conducted using the Student’s T-test. *p* < 0.05 indicates statistical significance.

## Supplementary Material

Supplemental Material

## Data Availability

The data presented in the manuscript are available at https://doi.org/10.6084/m9.figshare.25869082.v2.
