# Supplementary material for: A key role for P2RX5 in brown adipocyte differentiation and energy homeostasis
Source: Adipocyte. 2024 Nov 1;13(1):2421745. doi: 10.1080/21623945.2024.2421745 (PMC11540092; doi:10.1080/21623945.2024.2421745)
Supplement: Supplemental Material [file KADI_A_2421745_SM2538.docx]

**Supplementary Figure Legends**


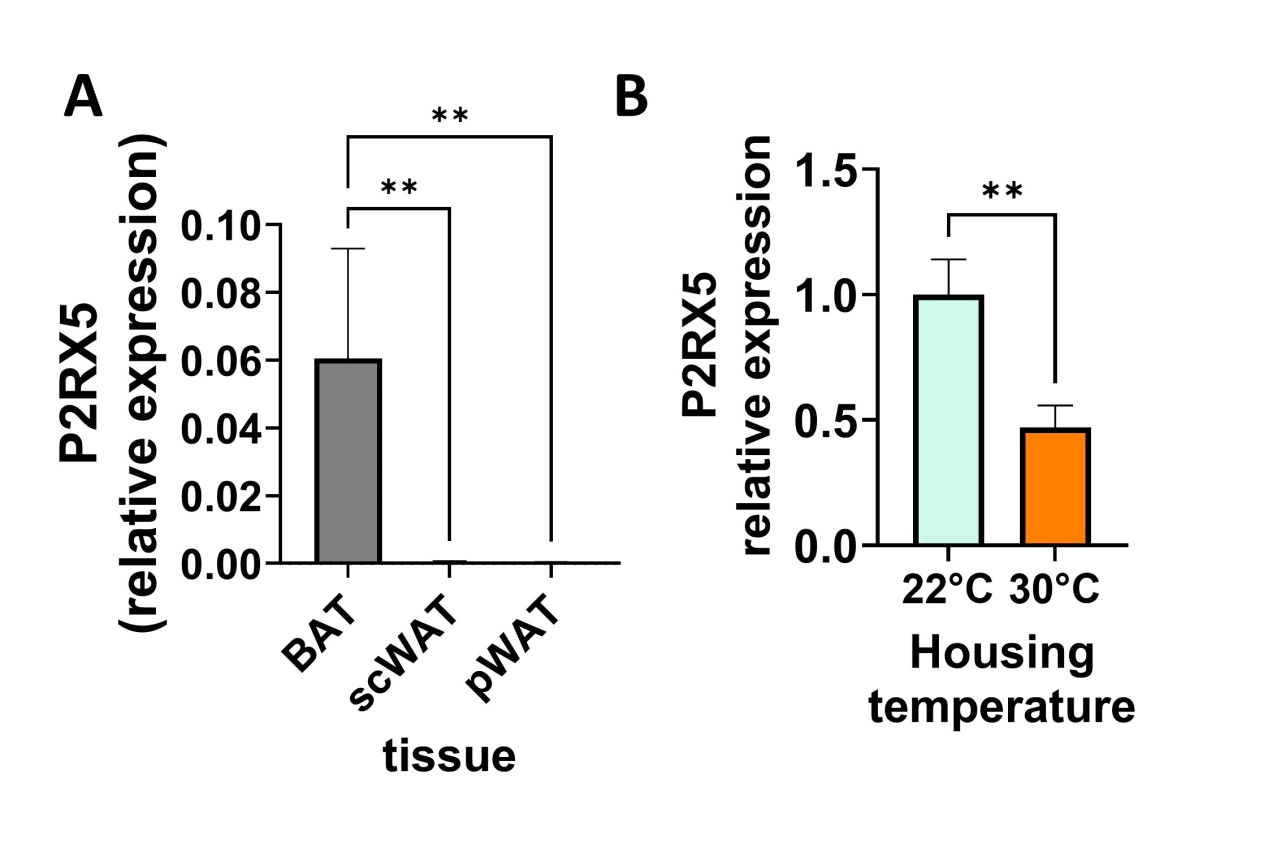


**Supplementary Figure 1 Characterization of P2RX5 in mouse adipose tissue, A**) P2RX5 expression in mouse fat pads (N=6): brown adipose tissue (BAT), subcutaneous white adipose tissue (scWAT) and perigonadal white adipose tissue (pWAT) (F(2,10=15.79, p<0.001). **B**) QPCR analysis of P2RX5 in mice housed at 22°C . *p<0.05,**p<0.01 (N=7-11).

**Alt-text:** Panel A: Bar graph showing P2RX5 relative expression in different tissues: BAT, scWAT, pWAT. Panel B: Bar graph comparing P2RX5 expression at 22°C and 30°C housing temperatures.


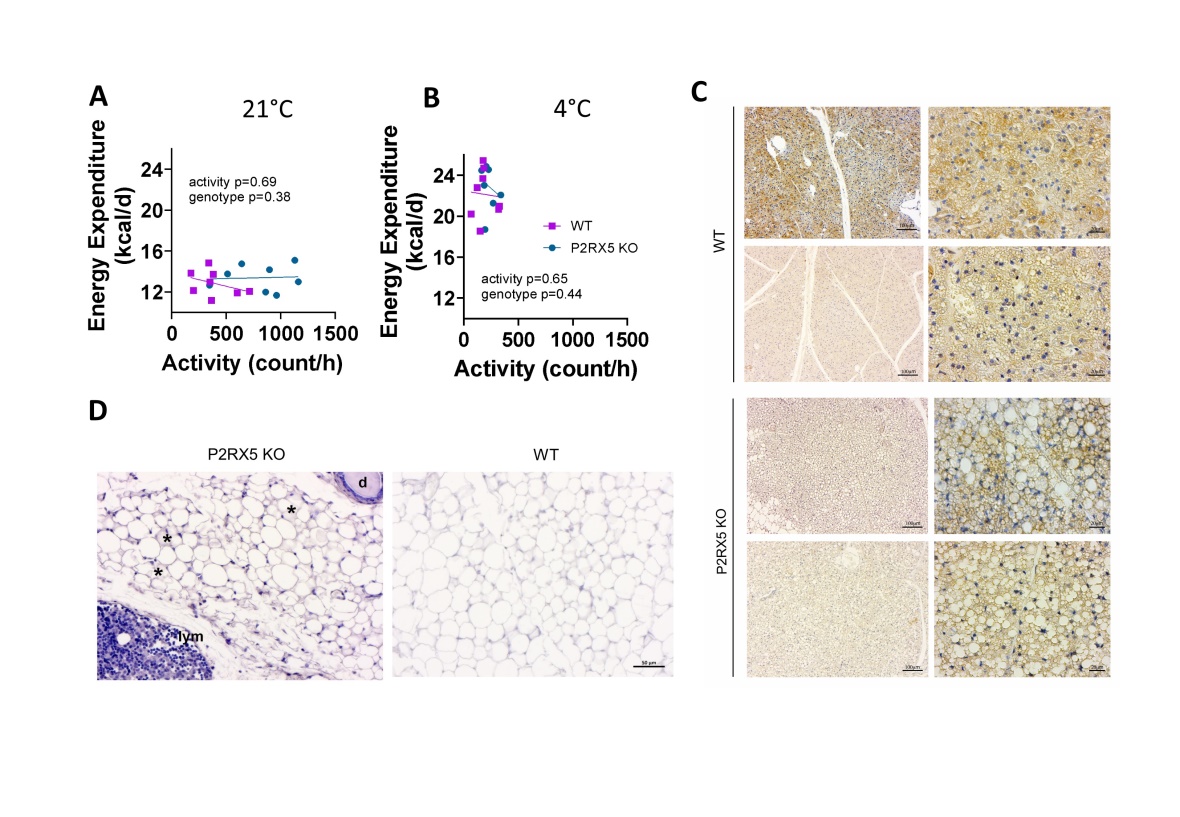


**Supplementary Figure 2** – Additional features of P2RX5 knock out (KO, n=8) and wild type (WT, n=8) mice during indirect calorimetry both during 24h at room temperature (**A**) and 4h at cold challenge (**B**) in relation to their activity levels. **C**) BAT of 2 individual WT and P2RX5 KO mice per genotype. Note that these are different from the animals presented in Figure 2B. Total N=3/group. **D**) Subcutaneous white adipose tissue histology in the peri-lympho-node area, disclosed the complete absence of multilocular brown-like adipocytes in P2RX5 knock out mice. Inguinal lympho-node (lym); duct (d); multilocular brown-like adipocytes (asterisks). Scale Bars in C and right panels in D = 50μM, Scale Bars in D left panels 100μM.

**Alt-text:** Panel A: Scatter plot of energy expenditure versus activity at 21°C. Panel B: Scatter plot of energy expenditure versus activity at 4°C. Panel C: Histological comparison of WT and P2RX5 KO tissues, various magnifications. Panel D: Histological images of P2RX5 KO and WT adipose tissues.


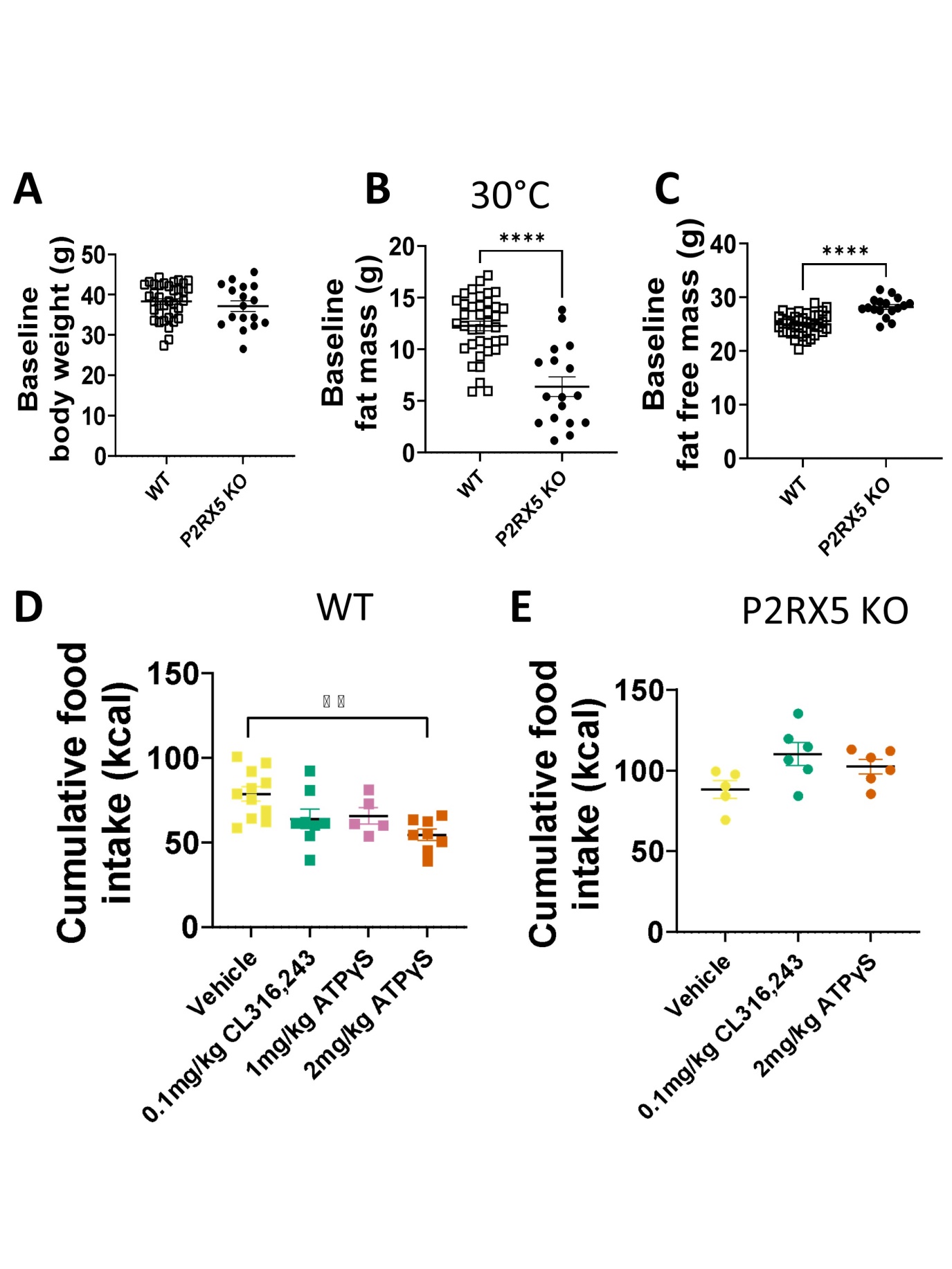


**Supplementary Figure 3 – Additional characterization of P2RX5 knock out (KO, n=5-6) and wild type (WT, n=5-11) mice at thermoneutrality (30±2°C). A-C**) Body composition characteristics at the end of the acclimation phase to thermoneutrality. **D-E**) Total food consumption during the pharmacological phase of the study for 13 days. **p<0.01, ****p<0.0001.

**Alt-text:** Panel A: Dot plot graph of baseline body weight (g) comparison between WT and P2RX5 KO mice. Panel B: Dot plot graph of baseline fat mass (g) comparison between WT and P2RX5 KO mice at 30°C. Panel C: Dot plot graph of baseline fat free mass (g) comparison between WT and P2RX5 KO mice. Panel D: Dot plot graph of cumulative food intake (kcal) in WT mice after the treatment with vehicle; 0.1 mg/kg CL316,243; 1 mg/kg ATPγS; 2 mg/kg ATPγS. Panel E: Dot plot graph of cumulative food intake (kcal) in P2RX5 KO mice after the treatment with vehicle; 0.1 mg/kg CL316,243; 2 mg/kg ATPγS.


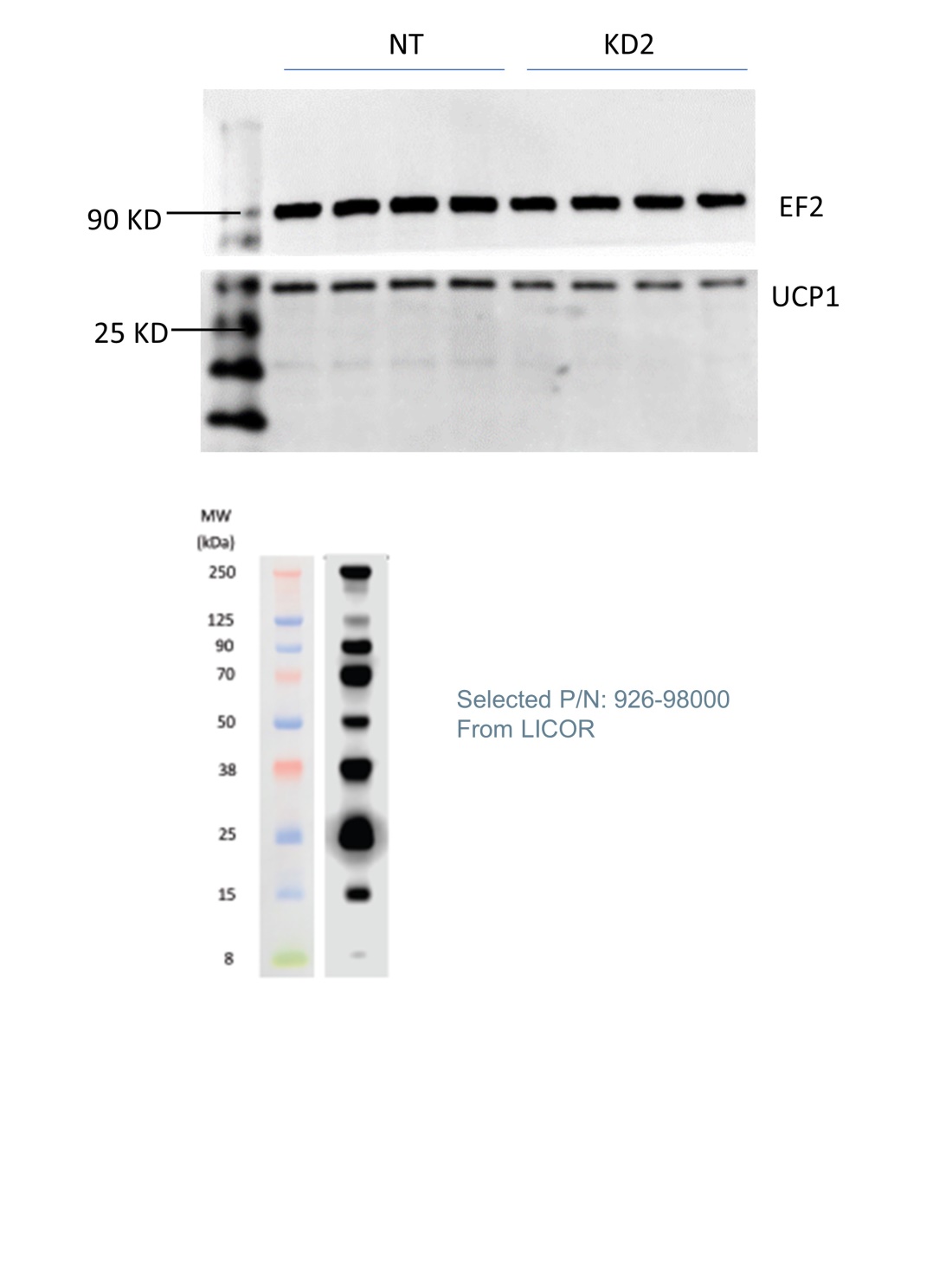


**Supplementary Figure 4.** Full blot image related to Figure 3H. The gel was cut in half and incubated with either EF2 or UCP1 antibodies based on their respective molecular weight. The ladder used in all western blot experiments is shown in the lower panel.

**Alt-text:** Western blot membranes with at 90 kDa EF2 and over 25 kDa UCP1, of 4 NT and 4 P2RX5 KD2 samples. Selected P/N from Licor.

**
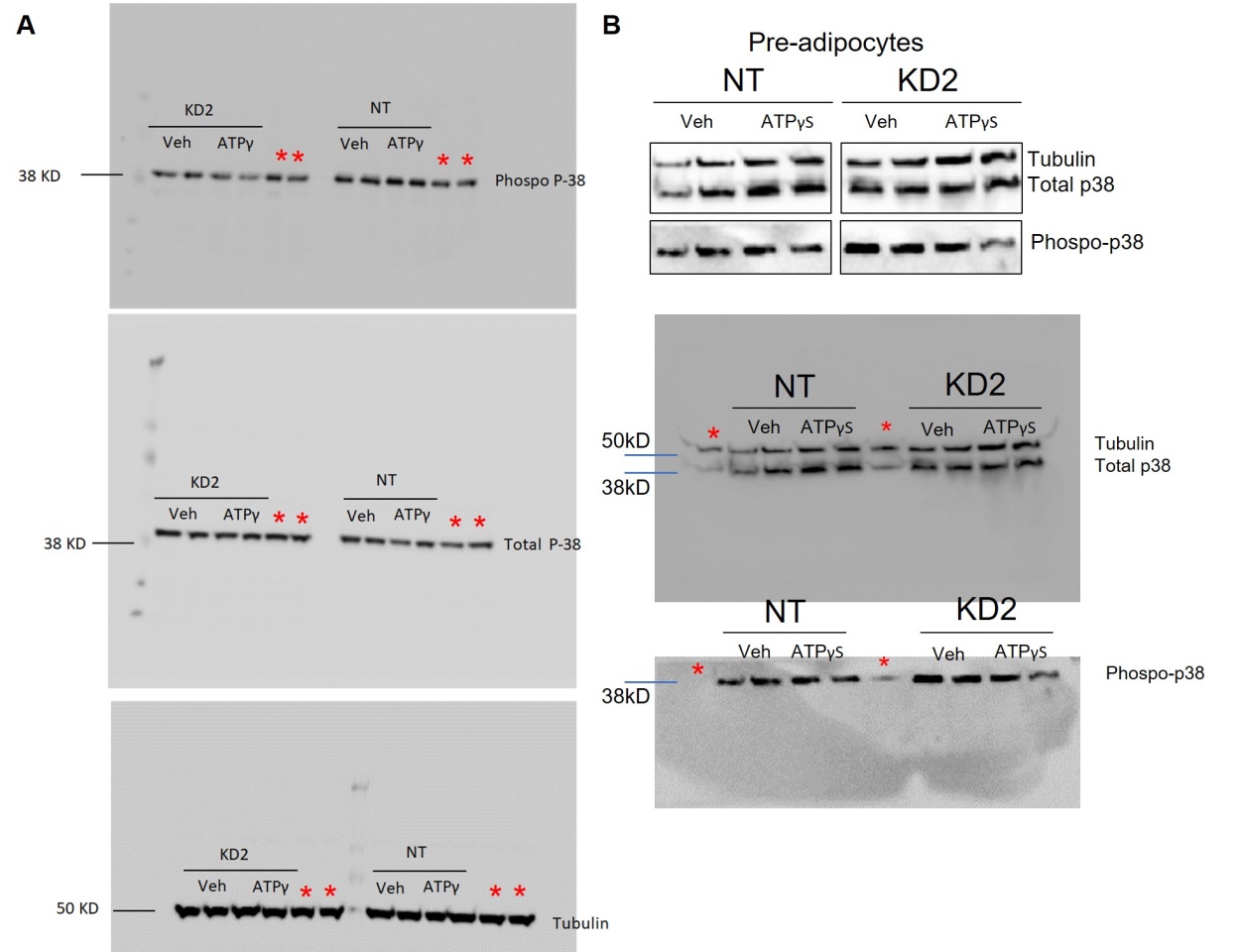
**

**Supplementary Figure 5.** Full blot image related to Figure 3I. **A**) First replicate. The last 2 lanes with a red asterisk are not included in the analysis because they are the result of a different treatment which was not included/discussed in this manuscript. **B**) Second replicate. The first lane with a red asterisk is not included in the analysis because it is the result of a different treatment which was not included/discussed in this manuscript. The ladder used is shown in Supplementary Figure 4.

**Alt-text:** Panel A: Western blot membranes of Phospho p-38 and Total p38 in vehicle and ATPγS in NT and P2XR5 KD shRNA pre-adipocytes, whit tubulin at 50 kDa as loading control.

Panel B: Western blot membranes of Phospho p-38 and Total p38 in vehicle and ATPγS in NT and P2XR5 KD shRNA pre-adipocytes whit tubulin at 50 kDa as loading control.


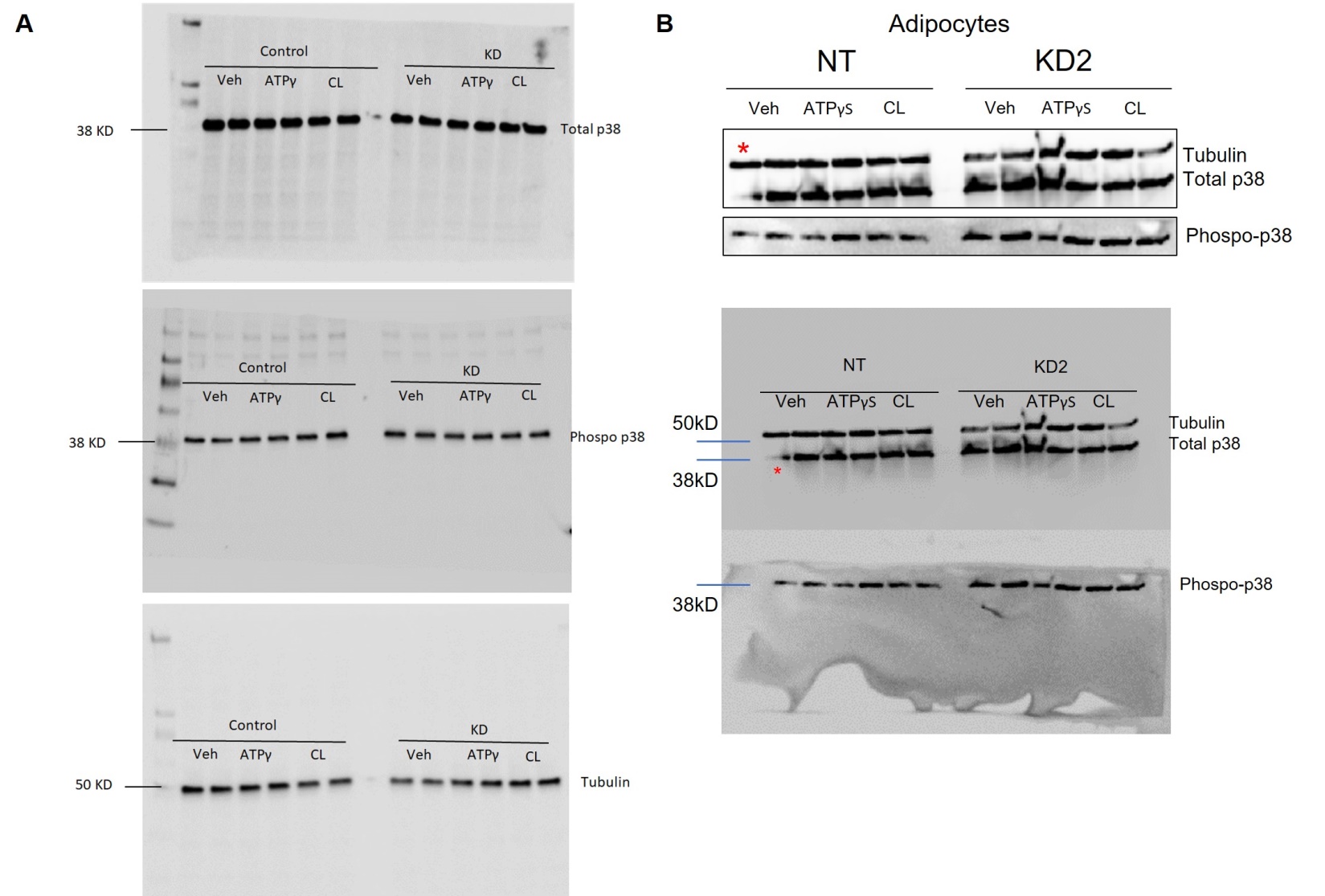


**Supplementary Figure 6.** Full blot image related to Figure 3J. **A**) First replicate. **B**) Second replicate. The lane with the red asterisk was excluded from the analysis because the total p38 lane was not stained properly for unknown reasons, resulting in a phosphorilated/total p38 >1. The ladder used is shown in Supplementary Figure 4.

**Alt-text:** Panel A: Western blot membranes of Phospho p-38 and Total p38 in vehicle and ATPγS and CL316,243 (CL) in NT and P2XR5 KD shRNA in adipocytes, whit tubulin at 50 kDa as loading control. Panel B: Western blot membranes of Phospho p-38 and Total p38 in vehicle and ATPγS in NT and P2XR5 KD pre-adipocytes whit tubulin at 50 kDa as loading control.


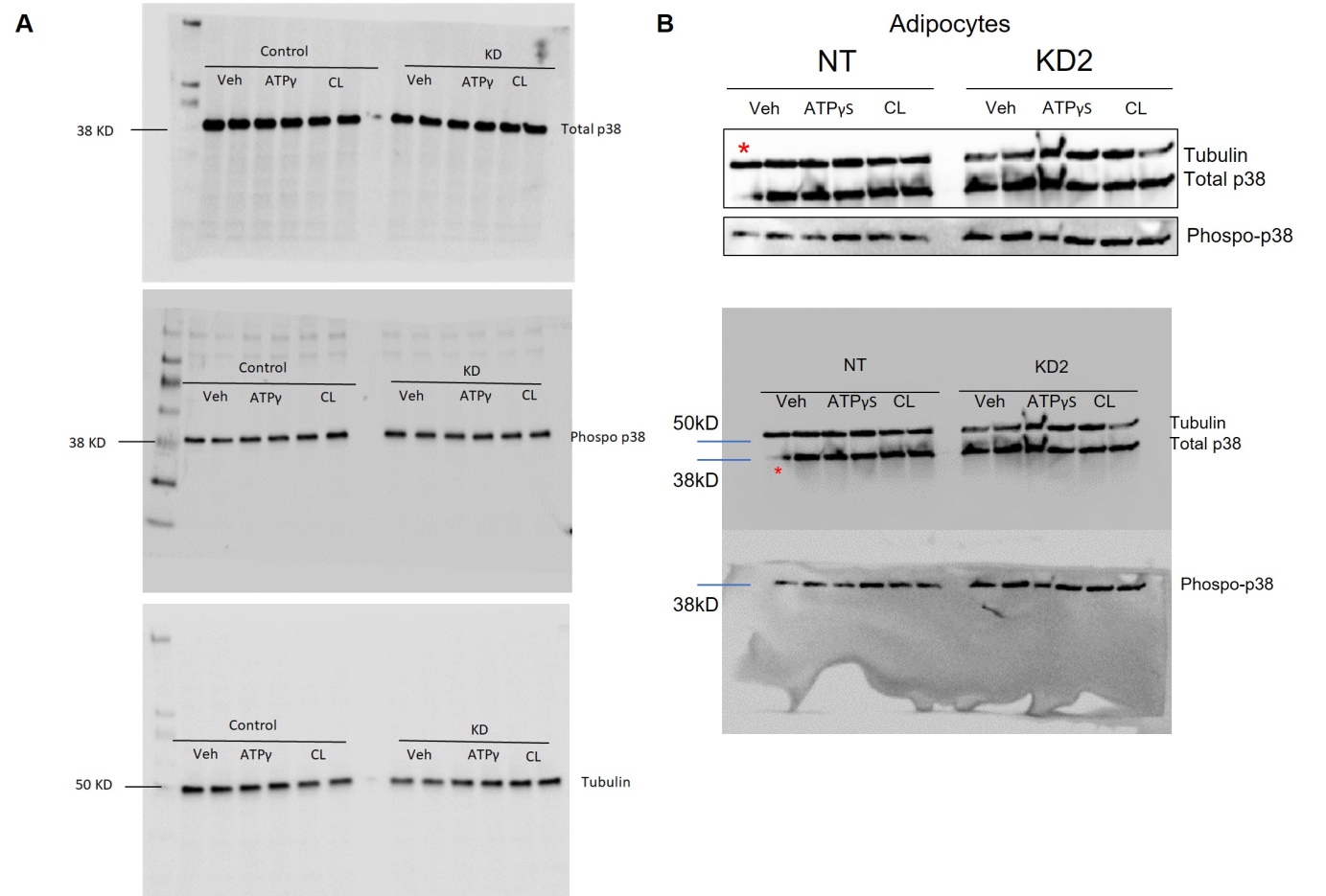


**Supplementary Figure 7 — Western Blot Images of Wild type and P2RX5 knock out mice at thermoneutrality (30±2°C). A**) Cropped and combined images for wild type mice with respective treatment groups highlighted. **B,C**) Full blot image related to panel A showing UCP1 (**B**) and beta-Tubulin (**C**). **D**) Cropped and combined images for P2RX5 knock out mice with respective groups highlighted. **E,F**) Full blot images related to panel D showing beta-Tubulin (Top bands, ~50kDa) and UCP1 (Bottom bands, ~37kDa). The gels in E and F were cut in half and incubated with either Tubulin or UCP1 antibodies based on their respective molecular weight. The ladder used is shown in Supplementary Figure 4.

**Alt-text:** Panel A: Arranged western blot membranes of UCP1 in saline, ATPγS1, ATPγS2 and CL316,243 in WT mice, whit tubulin at 50 kDa as loading control. Panel B: Original western blot membranes of UCP1 in saline, ATPγS1, ATPγS2 and CL316,243 in WT mice. Panel C: Original western blot membranes of tubulin in saline, ATPγS1, ATPγS2 and CL316,243 in WT mice. Panel D: Arranged western blot membranes of UCP1 in saline, ATPγS2 and CL316,243 in P2XR5 KD mice, whit tubulin at 50 kDa as loading control. Panel E: Original western blot membranes of UCP1 in saline, ATPγS2 and CL316,243 in P2XR5 KD mice, whit tubulin at 50 kDa as loading control. Panel F: Original western blot membranes of UCP1 in saline, ATPγS2 and CL316,243 in P2XR5 KD mice, whit tubulin at 50 kDa as loading control.
